# Reticulate Evolution in AA-Genome Wild Rice in Australia

**DOI:** 10.3389/fpls.2022.767635

**Published:** 2022-03-11

**Authors:** Sharmin Hasan, Agnelo Furtado, Robert Henry

**Affiliations:** ^1^Queensland Alliance for Agriculture and Food Innovation, The University of Queensland, Brisbane, QLD, Australia; ^2^Department of Botany, Jagannath University, Dhaka, Bangladesh

**Keywords:** hybridisation, introgression, chloroplast capture, reproductive barrier, reticulate evolution

## Abstract

The wild rice gene pool, i.e., AA-genome, in Australia is geographically and genetically distinct from that in Asia. Two distinct taxa are found growing together in northern Australia, *Oryza meridionalis* (including annual and perennial forms) and an *Oryza rufipogon* like taxa that have been shown to have a chloroplast genome sequence that is closer to that of *O. meridionalis* than to *O. rufipogon* from Asia. Rare plants of intermediate morphology have been observed in the wild despite a reported reproductive barrier between these two species. We now report the resequencing of plants from 26 populations including both taxa and putative hybrids. A comparison of chloroplast and nuclear genome sequences indicated re-combinations that demonstrated hybridisation in both directions. Individuals with intermediate morphology had high nuclear genome heterozygosity consistent with a hybrid origin. An examination of specific genes (e.g., starch biosynthesis genes) revealed the presence of heterozygotes with alleles from both parents suggesting that some wild plants were early generation hybrids. These plants may have low cross-fertility preserving the continuation of the two distinct species. Repeated backcrossing of these rare hybrids to one parent would explain the plants exhibiting chloroplast capture. These observations suggest that reticulate evolution is continuing in wild *Oryza* populations and may have been a key process in rice evolution and domestication.

## Introduction

Hybridisation and introgression undoubtedly play a crucial role in evolution allowing gene exchange between species resulting in genomic diversification and novel genetic combinations (Stebbins, 1959). Cytonuclear non-concordance, i.e., incongruence between organellar (plastid and mitochondrial) and nuclear genome phylogenies appears to reflect both hybridisation and successive introgression (Rieseberg, 1991; Nge et al., 2021). Recurrent hybridisation results in contrasting gene trees in two ways: (1) if recurrent unidirectional cytoplasmic geneflow from one species can invade another species in the absence of nuclear geneflow (Rieseberg and Wendel, 1993), and (2) if unidirectional nuclear geneflow but not cytoplasmic geneflow is allowed to invade a species (Petit et al., 2001). However, cytoplasmic genome invasion occurs in plants more frequently than nuclear genome introgression (Rieseberg and Wendel, 1993). Cytoplasmic geneflow results in chloroplast capture, which may occur due to partial cytoplasmic male sterility, nuclear genome incompatibilities, or partial selfing in species (Tsitrone et al., 2003). Contrasting gene trees can be attributed to sampling error, evolutionary convergence, evolutionary rate heterogeneity, lineage sorting, and reticulation (Rieseberg and Soltis, 1991). Chloroplast capture has been used to infer reticulate evolution in many plant species such as *Pisum* (Palmer et al., 1985), Australian cotton (Wendel et al., 1991), sunflower (Rieseberg, 1991), peonies (Sang et al., 1995), *Opuntia* (Griffith, 2003), soybeans (Doyle et al., 2003); kiwifruit (Chat et al., 2004), the wheat tribe (*Elymus*) (Mason-Gamer, 2004); baobabs (Karimi et al., 2019); and *Adenanthos* (Nge et al., 2021). Much effort has been put into re-constructing reticulate evolution by means of investigating putative hybrid origins through multiple molecular techniques (Vriesendorp and Bakker, 2005). Morphological features along with geographical, ecological, biosystematics, and cytological data have been used to infer hybrid origin. However, genetic evidence provides the greatest power of inference in detecting putative interspecific hybrid origin (Griffith, 2003). Genetic evidence can be derived through implying an additivity of molecular markers (Wendel et al., 1991; Griffith, 2003), polymorphic nucleotide additivity at a single position, i.e., ITS additivity (Sang et al., 1995), and incongruence between gene trees (Mason-Gamer, 2004). Next-generation sequencing (NGS) approaches appear to be increasingly effective in addressing the causes of cytonuclear incongruence (Lemmon et al., 2012; Weitemier et al., 2014).

Rice (the genus *Oryza*) belongs to the tribe Oryzeae—a member of the grass subfamily Ehrhartoideae. The genus *Oryza* comprises 11 genome groups of which the AA-genome group (with eight diploid species) is the most diverged group, distributed throughout the world, except for, Antarctica (Vaughan, 1989). The other genome groups are BB (one *Oryza* species), BBCC (three *Oryza* species), CC (three *Oryza* species), CCDD (three *Oryza* species), EE (one *Oryza* species), FF (one *Oryza* species), GG (three *Oryza* species), HHJJ (two *Oryza* species), HHKK (one *Oryza* species), and KKLL (one *Oryza* species) (Brar and Khush, 2018). Vicariance events and long-distance dispersal explain the evolution and divergence of AA genomes globally (Tang et al., 2010). The estimated mean divergence age of the AA genome from other *Oryza* genomes is 2.41 million years ago (Stein et al., 2018). Four *Oryza* species have been recognised in Australia (Henry et al., 2010). Of these, two wild *Oryza* species belong to the AA-genome clade: *Oryza meridionalis* Ng. and *Oryza rufipogon* Griff. The former was originally characterised by annual life history, self-pollination, and short anther length (Ng et al., 1981). More recently, the *O. meridionalis* gene pool has been shown to include both annual and perennial forms (Sotowa et al., 2013; Henry, 2019). *O. rufipogon* is considered a native perennial in Northern Queensland, Australia (Henry et al., 2010). Australian *O. rufipogon* populations have been shown to have a chloroplast genome that is closer to *O. meridionalis* than to *O. rufipogon* from Asia (Brozynska et al., 2014). This indicates that the Australian population might need to be considered as separate taxon (Brozynska et al., 2017).

Reproductive barriers appear to be common in the AA genome *Oryza* species, especially in crosses between *O. sativa* and its wild relatives (including *O. meridionalis*), resulting in hybrid sterility (Li et al., 2007). Hybridisation between *O. meridionalis* and Asian wild rice produced low fertility hybrids (Naredo et al., 1997). Isolation due to pollen sterility was identified in *O. sativa* L. × *O. meridionalis* hybrids, resulting in partial and full abortion of male gametes (Li et al., 2018). Post-zygotic reproductive isolation happens to abort seeds in first filial generation (F1) hybrid offspring derived from hybridisation between *O. meridionalis*, and *O. sativa* (Toyomoto et al., 2019). Furthermore, geographic distributions and differences in flowering times influence intra-and interspecific crossability of *O. meridionalis* in Australia (Juliano et al., 2005).

Cytonuclear discordant in phylogenetic trees (i.e., chloroplast capture) has been observed in these wild rice populations, which were assumed to be due to hybridisation between the *O. meridionalis* and *O. rufipogon* type taxa (Moner et al., 2018). These wild hybrid plants were identified in the field due to their intermediate morphological characteristics with combinations of spike morphology and anther size, suggesting the inheritance of traits from both parental taxa. However, the exact nature of their hybrid origin was not characterised by nuclear genome analysis.

We have now reexamined the origins of these hybrids by re-sequencing and analysing the nuclear genomes of these wild rice populations including wild plants of hybrid appearance.

## Materials and Methods

### Sample Collection

**FIGURE 1:**
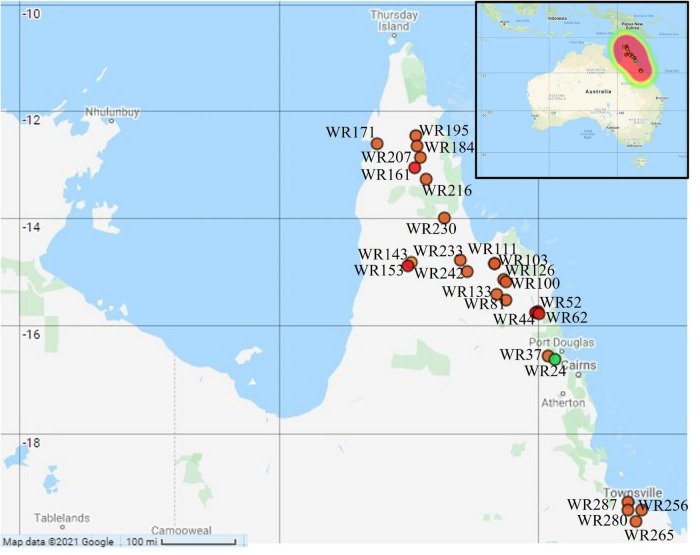
Geographical locations of the collected Australian wild rice samples: orange dot = *Oryza meridionalis*; green dot = *Oryza rufipogon* type taxa; red dot: hybrid. Map created with Map Maker at https://maps.co (Map Maker, 2021).

Australian wild rice is widely distributed from the south of Townsville to the northern parts of Cape York Peninsula, Queensland, Australia. Fresh leaves of wild rice from 26 different geographical sites were collected in the two consecutive years 2015 and 2016 by Moner et al. (2018) (Figure 1). Only one sample was collected from each site. Among these, 22 samples were collected in 2015 from 22 different sites in the northern parts of Cape York Peninsula while only four samples were collected in 2016 from the south of Townsville. The samples were kept in the dry ice all the way from the sampling sites to the laboratory until stored at −80°C freezer.

### DNA Extractions, Sequencing, and Quality Filtering

Fresh leaves were pulverised with tissue lyser (Qiagen, United States). Samples were kept in liquid nitrogen to prevent thawing throughout the pulverisation process. DNA was extracted from pulverised leaf tissue of wild rice samples following the CTAB method (Furtado, 2014). The quality and quantity of the extracted DNA were evaluated with a NanoDrop spectrophotometer (Thermo Fisher Scientific, Delaware, United States) as well as by electrophoresis on a 0.7% agarose gel stained with SYBR safe. The 260/280 nm ratio for 26 samples ranged from 1.11 to 2.06. Nextera DNA Flex Libraries were prepared. Nextera DNA Library Preparation Kits were designed in an automation-compatible workflow that introduced tagmentation chemistry, which joined DNA fragmentation and adapter ligation steps into a single 15-min reaction. Then these libraries were sequenced on a NovaSeq 6000 SP and S4 Flow cell along with other samples to produce 2 × 150 paired-end reads with a data yield of 20x whole genome coverage on average. A quality check was applied to the raw data using CLC Genomic Workbench (CLC-GWB) version 20 software (QIAGEN CLC Genomics Workbench 20, Denmark)^1^ to identify any sequencing error before performing further analysis. Sequences were trimmed at both 0.05 and 0.01 quality scores to truncate low-quality reads. Based on the percentage of loss of reads and bases, 0.01 trimmed reads were considered for further analysis. The total Illumina raw reads for the 26 samples ranged from 56,349,078 to 104,325,654 bp. The total raw nucleotides ranged between 8,416,673,405 and 15,628,960,467 bp. After trimming, the total 0.01 trimmed reads ranged from 54,631,067 to 98,924,768 and nucleotides from 7,925,007,230 to 14,260,593,039 bp (Supplementary Table 1).

### Illumina Reads Mapping and Genome-Wide Variant Detection

Prior to final mapping, the best mapping parameters were evaluated with the three different alignment quality thresholds: 1.0 length fraction (LF) and 0.95 similarity fraction (SF), 1.0 LF and 0.90 SF and 1.0 LF and 0.85 SF. Illumina paired-end reads were then mapped to the reference genome Os-Nipponbare-Reference-IRGSP-1, which was downloaded at Rice Annotation Project Database (Kawahara et al., 2013) with these three settings. The average mapped read depth (times of reference length), mapped reads (%), mapped bases (%), and total consensus length as a % of reference length were calculated from the output of mapped reads to make a comparison among the three different settings. After comparison, the 1 LF and 0.9 SF settings were found to be stringent enough to consider for final mapping. A total of 26 wild rice Illumina sequences quality trimmed at 0.01 (Phred equivalent > 20, 94–98% of the reads had a phred score greater than 35, average across all bases) was mapped to the Os-Nipponbare-Reference-IRGSP-1, separately using the tool “Map Reads to Reference” in CLC-GWB, with the following parameters: masking mode = no masking; match score = 1; mismatch cost = 2; cost of insertions and deletions = linear gap cost; insertion cost = 3; deletion cost = 3; length fraction = 1; similarity fraction = 0.9; global alignment = no; auto-detected paired distances = yes; non-specific match handling = map randomly. Mapped reads was subjected to variant analysis using the tool “Basic Variant Analysis” in CLC-GWB, with the following parameters: ploidy = 2; ignore positions with coverage above = 100,000; restrict calling to target regions = not set; ignore broken pairs = no; ignore non-specific matches = no; minimum coverage = 10; minimum count = 3; minimum frequency (%) = 25; neighborhood radius = 5; minimum central quality = 20; minimum neighborhood quality = 15; read direction filter = no; read position filter = no; remove pyro-error variants = no. Total variants that included SNV (single nucleotide variant), insertions, deletions, MNV (multiple nucleotide variants), and replacement were determined. Percentage of SNV and InDels (insertions and deletions) were calculated as: (total number of SNV/InDels × 100)/total number of variants detected (1). Heterozygous SNV positions were determined by SNV loci with two alleles. Heterozygosity across the whole genome was calculated as: (total heterozygous SNP position × 100)/the whole genome size (2). Unique heterozygous SNPs were those that were distinctively located in the reads of a sample. The whole-genome size of *O. rufipogon* type taxa (= Taxon A) and *O. meridionalis* (= Taxon B) was 384.8 and 354.9 Mb, respectively (Brozynska et al., 2017). Other data resources for *O. rufipogon* and *O. meridionalis* are available at (https://oryza-ensembl.gramene. org/Oryza_meridionalis/Info/Index, https://oryza-ensembl. gramene.org/Oryza_rufipogon/Info/Index, http://viewer.shigen. info/oryzagenome21detail/downloads/index.xhtml;jsessionid=16 d6389e9b7080a8c4fb7378b753). Sequence data for the Australian *O. rufipogon* like taxon is reported in Brozynska et al. (2017).

### Nuclear Geneflow and Genetic Structure Analysis

In further experiment, five natural hybrids (WR44, WR52, WR62, WR153, and WR161) were investigated to test for nuclear introgression from the putative parental populations. Among these, WR44, WR52, and WR153 were reported by Moner et al. (2018). The earlier study also identified two hybrids (WR65 and WR162), which were collected from Lakeland Cook town road and Merluna site 1, respectively. Two hybrids (WR62 and WR161), explored in this study, were also collected from the same sites but not reported as hybrids by Moner et al. (2018). These were collected from the same sites as WR65 and WR162, respectively. To ensure the chloroplast genome of WR62 and WR161 were of hybrid origin showing similarity with the previously published samples WR65 and WR162 (Moner et al., 2018), respectively, 0.01 trimmed data of WR62 and WR161 were mapped to the reference chloroplast genome of *O. meridionalis*, and the reference chloroplast genome of *O. rufipogon* type taxa obtained from Brozynska et al. (2014). Mapping was done following the reference-based mapping workflow explained by Moner et al. (2018) using CLC-GWB. Then the consensus sequences of the chloroplast genome were extracted from these two samples (sequences S1 and S2 provided as Supplementary Material). Chloroplast genomes of seven samples; WR62, WR161 from this study, WR65 and WR162 (Moner et al., 2018), two reference chloroplast genomes of *O. meridionalis* and *O. rufipogon* type taxa (Brozynska et al., 2014), and *Oryza officinalis* Wall. ex Watt (Moner et al., 2018) were aligned with Multiple Alignment using Fast Fourier Transform (MAFFT) version 7.407 (Katoh and Standley, 2013) with default parameters in Geneious version 11.1.5 software.^2^ The substitution model for the phylogenetic analysis was selected by the jModelTest2 (Darriba et al., 2012) on the Extreme Science and Engineering Discovery Environment (XSEDE). The parameters for best selected model following the Akaike information criterion (AIC) was used in maximum likelihood tree construction: Model = TVM+I+G, partition = 012314, −lnL = 1888839.3939, *K* = 21, feqA = 0.3068, freqC = 0.1941; feqG = 0.1957; freqT = 0.3034; ti/tv = −; p-inv = 0.897; R(a) = 1.0604; R(b) = 2.5331, R(c) = 0.5698; R(d) = 0.9914; R(e) = 2.5331; R(f) = 1; gamma = 0.587. Phylogenetic reconstruction was performed using PAUP* version 4 software (Swofford, 2003) with maximum likelihood (ML) with heuristic searching, retain group with frequency > 50%, bisection, reconnection branch swapping method and 1,000 bootstrap replicates. *O. officinalis* was selected as an outgroup species in the phylogenetic tree.

Single nucleotide polymorphism (SNP) genotypes of six genes located on chromosomes 4, 6, and 7 from hybrids and putative parental taxa were aligned, with respect to Os-Nipponbare-Reference-IRGSP-1 with CLC-GWB to screen for contributing donor parents in genotypes of hybrid populations. These genes include *seed shattering 4* (*sh4*) on chromosome 4, four starch synthesis related genes on chromosome 6 [*granule bound starch synthase I* (*GBSSI*), *soluble starch synthase-I* (*SSI*), *starch branching enzyme I* (*SBEI*), and *alkali degeneration* (*SSIIa* or *ALK*)] and *granule bound starch synthase II* (*GBSSII*) on chromosome 7. Plausible hybridisation generations were determined based on the level of heterozygosity across the whole genome of these hybrids.

Four taxon (ABBA-BABA) test as a measure of geneflow was performed with Hybrid Check version 1 software (Ward and van Oosterhout, 2016). Four genes (*GBSSI*, *SSI*, *SBEI*, and *ALK)* from two putative parental populations (WR24 and WR81), one hybrid and outgroup species were first aligned with MAFFT version 7.407 (Katoh and Standley, 2013) with default parameters in Geneious version 11.1.5 software to generate an aligned sequence. Then, an aligned sequence was used to perform the four-taxon test with a topology (((P1, P2), P3) A), where a triplet comprises of a set of three aligned sequences from a hybrid (P3) and two putative parental populations (P1 and P2). *O. sativa* spp. *japonica* cv. Nipponbare was considered an outgroup species (A). We simulated a total of 5 four-taxon tests to determine biparental nuclear geneflow between hybrid and two putative parental taxa. We estimated Patterson’s *D*-statistic based on the random distribution of ABBA-BABA polymorphisms in a four-taxon test (Martin et al., 2015). The *D*-statistic estimates the deviation from the distribution of ABBA-BABA polymorphisms in a four-taxon tree. If *D* > 0, introgression occurs between P2 and P3, and if *D* < 0, introgression occurs between P1 and P3. We performed a block jackknife method using a block length of 1,000 bp to calculate *Z* score and *p*-value to test significant deviation from the null expectation (*D* = 0, lineage sorting).

Genome-wide SNP data from 26 wild rice samples were used to perform principal component analysis (PCA) to assess the genetic variation in wild rice populations using plink version 1.9 (Purcell et al., 2007). To ensure the SNP data was not biased with any linkages, linkage disequilibrium (LD) based pruning was done to generate a final neutral SNP data for PCA analysis. LD pruning was performed using the “–indep-pairwise” option by specifying a window of 50 kb, with a step window size of 10 bp and a threshold of linkage, *r*^2^ = 0.2 (which recursively eliminates SNPs within the sliding window if *r*^2^ is greater than 0.2). To investigate the relationship of population structure with geographic distance, a subset of genome-wide SNP data (2,987,364 SNPs of 12,247,310 SNPs) from 26 wild rice genotypes (two alleles per loci) were used to perform isolation by distance (IBD) analysis using Mantel test (Mantel, 1967) with the R package adegenet version 2.0.1 (Jombart and Ahmed, 2011). Nei (1972) pairwise genetic distances vs. geographic distances were correlated with the function mantel.randtest using a Monte Carlo simulation with 999 permutations in IBD analysis.

## Results

### Mapping Outputs and Sequence Variation

Sequence variation from *O. sativa* was substantial among the 26 samples (Supplementary Table 1). The average sequence read depth (times reference length) ranged from 20.7 in WR153 to 46.9 in WR103 (Supplementary Table 1). While an average mapped sequence read depth (times reference length) varied from 8.88 in WR24 to 46.7 in WR103. The percentage of mapped reads was highest in WR44 (72.0%) and the lowest in WR24 (24.0%). Similarly, the highest and lowest percentage of mapped bases were 71.9 and 23.8% in WR44 and WR24, respectively. The total consensus sequences length ranged from 257,716,289 bp in WR37 to 322,074,565 bp in WR24. The total consensus as a percentage of the reference ranged from 67.4% in WR37 to 83% in WR44. The data for eight samples were also mapped to the lower quality draft genomes (Brozynska et al., 2017) for the two Australian taxa (Supplementary Table 2) resulting in somewhat higher genome coverage. WR 24 displayed an *O. rufipogon* type nuclear genome and showed much greater coverage of this reference confirming it was closer to *O. sativa* than the *O. meridionalis* types. Further analysis was based upon the use of the common high-quality *O, sativa* genome to align the reads to explore hybridisation and heterozygosity in the wild populations.

### Chloroplast and Nuclear Genome Characterisations

The analysis of phylogenetic relationships based on the whole chloroplast and nuclear genome had suggested that chloroplast capture was present in Australian wild rice populations (Moner et al., 2018). The whole chloroplast and nuclear genome of both *O. rufipogon* type taxa and *O. meridionalis* were compared with the chloroplast and nuclear genome of WR62 and WR161 to determine the origin of these samples that had not been characterised in the earlier study of Moner et al. (2018). The results confirmed that these were of hybrid origin as the chloroplast genome of WR62 was grouped with the *O. meridionalis* type (Brozynska et al., 2014) while the nuclear genome was of the *O. rufipogon* type taxa (Supplementary Figure 1). While WR161 contained an *O. meridionalis* type nuclear genome and an *O. rufipogon* type chloroplast genome (Brozynska et al., 2014). This reinforced the presence of bidirectional chloroplast capture in hybridising populations in the Australian wild rice gene pool. Hybrids acquired their chloroplast genome from either the *O. rufipogon* type or *O. meridionalis* during hybridisation. The other three hybrids (WR44, WR52, WR153) contained an *O. rufipogon* type chloroplast genome and an *O. meridionalis* type nuclear genome.

### Analysis of Gene Loci

**FIGURE 2:**
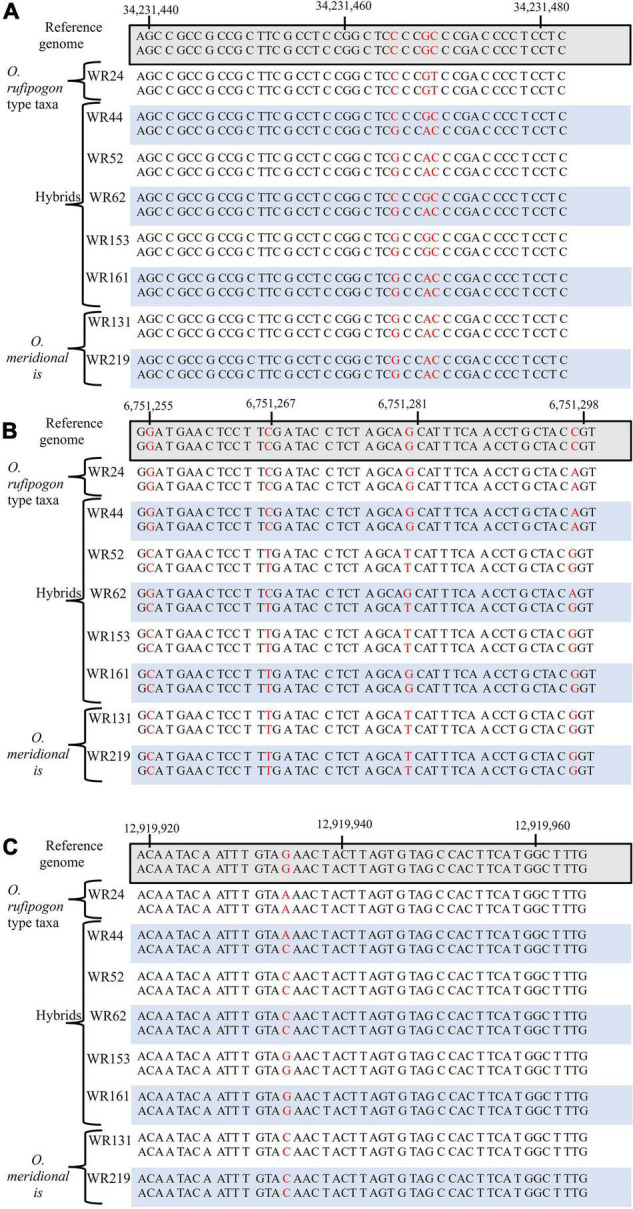
Alignment of single nucleotide polymorphism (SNP) genotypes of *O. rufipogon* type taxa (WR24), hybrids (WR44, WR52, WR62, WR153, and WR161) and *O. meridionalis* (WR133 and WR219), in relation to, *O. sativa* spp. *japonica* cv. Nipponbare as a reference genome: **(A)** *seed shattering 4* (*sh4*) gene on chromosome 4, **(B)** *alkali degeneration* (*ALK*) gene on chromosome 6, and **(C)** *granular bound starch synthase II* (*GBSSII*) on chromosome 7. The red color denotes SNPs.

SNP were analysed for six genes on chromosomes 4, 6, and 7 were analysed to assess the genetic contribution of the putative parents to the natural hybrid populations in relation to *O. sativa* spp. *japonica* cv. Nipponbare reference genome. Of the six genes, one gene (*sh4*) on chromosome 4 is associated with seed shattering while the other five genes are starch synthesis-related genes located on chromosome 6 (*GBSSI*, *SSI*, *SBEI*, and *ALK*) and chromosome *7* (*GBSSII*). From SNP data, it was clearly shown that the six genes of WR62 received alleles from both *O. rufipogon* type taxa and *O. meridionalis* parents. *SBEI* and *GBSSII* had heterozygous SNP genotypes with both *O. rufipogon* type taxa and *O. meridionalis* alleles in all of five hybrids (Table 1 and Figure 2). However, the *sh4* alleles were heterozygous for WR44, WR62, and WR161 and homozygous for WR52 and WR153. Two hybrids (WR153 and WR161) also had heterozygous loci for *ALK* and *SSI*, respectively. Sequence alignment images of SNP genotypes of sh4 gene (ID: Os04g0670900-1, position on chromosome 4 from 34,231,186 to 34,233,221 bp), *ALK* gene (ID: Os06g0229800-1, position on chromosome 6 from 6,748,398 to 6,753,302 bp), and *GBSSII* (ID: Os07g0412100-1, position on chromosome 7 from 12,916,883 to 12,924,202 bp) (RAPDB, 2021) showed heterozygous locus as in Figure 2. WR44 and WR62 showed heterozygous loci at the 34,231,465 and 34,231,468 base positions (Figure 2A) while WR62 and WR44 showed heterozygous loci at 6,751,298 and 12,919,934 base position, respectively (Figures 2B,C). In all cases, a heterozygote was formed by receiving an allele from *O. rufipogon* type taxa and the alternative allele from *O. meridionalis*. Hybrids with homozygous loci represented alleles mostly from *O. meridionalis*. The number of total variants across the whole genome varied among hybrids, ranging between 5,484,261 and 8,024,717. W44 and W62 are early generation hybrids as shown by the presence of alleles from both parents while others are backcrossed to recover a nuclear genome similar to the recurrent parent. The highest number of SNPs (6,910,977) was found in WR44, followed by 5,917,274 in WR62, 5,291,708 in WR52, 5,295,508 in WR161 and 4,764,266 in WR153 (Table 1). Similarly, the highest number of heterozygous SNPs were found in WR44 (2,464,823) and the lowest number in WR153 (1,111,890). To determine the heterozygosity across the whole genome, the genome size of *O. rufipogon* type taxa was applied to the above-mentioned formula (2) for WR62, which exhibited *O. rufipogon* type nuclear genome while the genome size of *O. meridionalis* was used for the other hybrids. A high level of genetic variation was observed in both WR44 (0.69%) and WR62 (0.6%) across the whole genome followed by WR161 (0.34%), WR52 (0.32%), and WR153 (0.31%) in relation to *O. sativa* spp. *japonica* cv. Nipponbare (Table 1). The level of heterozygosity across the whole genome of these hybrids confirmed hybridisation events and led to the conclusion that WR44 and WR62 were early generation hybrids while the other hybrids were much later generations due to recurrent backcrossing with one parent (*O. meridionalis*).

**TABLE 1:** Characterisation of natural hybrids in wild rice populations.

Hybrid	SNP genotypes of six genes on chromosome 4, 6, and 7						Total SNPs	Total heterozygous SNPs	Heterozygosity across the whole genome (%)	Plausible hybrid generations
	*sh4 alleles*	*GBSSI* alleles	*SSI* alleles	*SBEI* alleles	*ALK alleles*	*GBSSII alleles*				
WR44	AB	BB	BB	AB	BB	AB	6,910,977	2,464,823	0.69	Early (F1/F2)
WR52	BB	BB	BB	AB	BB	AB	5,291,708	1,137,527	0.32	Later
WR62	AB	AB	AB	AB	AB	AB	5,917,274	2,318,878	0.60	Early (F1/F2)
WR153	BB	BB	BB	AB	AB	AB	4,764,266	1,111,890	0.31	Later
WR161	AB	BB	AB	AB	BB	AB	5,295,508	1,189,627	0.34	Later

*Plausible hybrid generations are shown based on the level of heterozygosity across the whole genome. AB and BB denote heterozygous and homozygous loci, respectively.*

### Analysis of Genetic Introgression

A four-taxon test (as *D*-statistic) was performed to test for biallelic mutation patterns (ABBA-BABA) to determine geneflow between the hybrids and two putative perennial parental populations in a topology {[(*O. rufipogon* type taxa, *O. meridionalis*), hybrid], *O. sativa* spp. *japonica* cv. Nipponbare}. The length of a MAFFT aligned sequence was 4,473 bp with a total of four blocks: (1–1,118); (1,119–2,236); (2,237–3,354); and (3,355–4,473). The *D*-statistic value based on the distribution of AABB and BABA on each block showed significant deviation for all four-taxon trees (Table 2). The negative *D*-statistic value suggested potential genetic introgression between *O. rufipogon* type taxa (WR24) and hybrid while the positive value suggests geneflow between *O. meridionalis* (WR81) and hybrid. In all cases, *O. sativa* spp. *japonica* cv. Nipponbare was an outgroup. The *Z-*score for all four-taxon tests was significant (*p* < 0.001).

**TABLE 2:** Estimation of geneflow in a triplet species following four-taxon tests with HybridCheck version 1.0 software.

Species in triplet			Outgroup species	Block start	Block end	numABBA	numBABA	*D*-statistic	*Z*-score	Direction of geneflow
P1	P2	P3	A							
WR24	WR81	WR44	Nipponbare	1	1,118	168	10	0.47	0.35*	P3→P1
WR24	WR81	WR52	Nipponbare	1	1,118	133	6	0.38	−0.38*	P3→P1
WR24	WR81	WR62	Nipponbare	1	1,118	66	18	0.06	−1.81*	P3→P1
WR24	WR81	WR153	Nipponbare	1	1,118	73	18	0.03	−2.14*	P3→P1
WR24	WR81	WR161	Nipponbare	1	1,118	55	0	0.33	−0.78*	P3→P1
WR24	WR81	WR44	Nipponbare	1,119	2,236	0	8	–0.28	0.35*	P2→P3
WR24	WR81	WR52	Nipponbare	1,119	2,236	0	13	–0.49	−0.38*	P2→P3
WR24	WR81	WR62	Nipponbare	1,119	2,236	0	27	–0.69	−1.81*	P2→P3
WR24	WR81	WR153	Nipponbare	1,119	2,236	0	15	–0.49	−2.14*	P2→P3
WR24	WR81	WR161	Nipponbare	1,119	2,236	0	14	–0.43	−0.78*	P2→P3
WR24	WR81	WR44	Nipponbare	2,237	3,354	9	19	–0.57	0.35*	P2→P3
WR24	WR81	WR52	Nipponbare	2,237	3,354	9	16	–0.65	−0.38*	P2→P3
WR24	WR81	WR62	Nipponbare	2,237	3,354	9	19	–0.66	−1.81*	P2→P3
WR24	WR81	WR153	Nipponbare	2,237	3,354	9	19	–0.68	−2.14*	P2→P3
WR24	WR81	WR44	Nipponbare	3,355	4,472	19	21	–0.06	0.35*	P2→P3
WR24	WR81	WR52	Nipponbare	3,355	4,472	19	21	–0.12	−0.38*	P2→P3
WR24	WR81	WR62	Nipponbare	3,355	4,472	19	21	–0.16	−1.81*	P2→P3
WR24	WR81	WR153	Nipponbare	3,355	4,472	19	21	–0.31	−2.14*	P2→P3
WR24	WR81	WR161	Nipponbare	3,355	4,472	19	21	–0.14	−0.78*	P2→P3

Triplets are characterised: P1 = O. rufipogon type taxa; P2 = O. meridionalis; and P3 = Hybrid. O. sativa spp. japonica cv. Nipponbare (A) is an outgroup species. The length of aligned nucleotide is 4,473 bp containing four blocks: (1–1,118); (1,119–2,236); (2,237–3,354); and (3,355–4,473). The biallelic patterns based on the number of ABBA-BABA in a triplet used in the Patterson’s D-statistic are shown. P3→P1 denotes geneflow between O. rufipogon type taxa and hybrid while P2→P3 denotes geneflow between O. meridionalis and hybrid. *Z-score represents statistically significant at p < 0.001.

### Intra- and Interspecific Variations in the Wild Rice Gene Pool

To estimate intra- and interspecific variations in the wild rice gene pool, variants comprised of five different types: SNV, insertions, deletions, MNV, and replacement were assessed across the whole genome of twenty-one putative parental taxa (including one *O. rufipogon* type taxa genotypes and 20 *O. meridionalis* genotypes). The number of allelic positions where the variants were located was determined. The total number of variants ranged from 1,918,262 in WR24 to 7,310,928 in WR81 (Table 3). Overall, 87–90% of the total variants in an individual were SNPs for all samples. While the proportion of InDels (insertions and deletions) was low ranging between 6 and 8%. The number of heterozygous SNP positions varied among wild rice populations. The highest number of heterozygous SNP was found in WR81 (1,365,802) while the lowest number of heterozygous SNP was found in WR24 (632,272). Consequently, the level of heterozygosity across the whole genome was higher in *O. meridionalis* (0.38%), and the least heterozygosity was found in *O. rufipogon* type taxa (0.16%). The SNPs that were uniquely present in individuals were determined to define the genetic relationships between individuals. The rare or unique heterozygous SNP position (%) ranged from 4 to 48%. The highest number of unique heterozygous SNP positions was in *O. rufipogon* type taxa and the lowest number in *O. meridionalis*, indicating *O. rufipogon* type taxa might be subject to more genetic drift compared to *O. meridionalis* populations due to their smaller population size.

**TABLE 3:** Genetic variation across the whole genome of 21 wild rice samples, excluding hybrids.

Species	Samples	Total number of variants	SNP (%)	InDel (%)	Total number of heterozygous SNV position	Heterozygous SNP variation across the whole genome (%)	*Unique heterozygous SNP (%)
*O. rufipogon* type taxa	WR24	1,918,262	90	6	632,272	0.16	48
*O. meridionalis*	WR37	3,745,572	87	8	827,689	0.23	6
	WR81	7,310,928	87	8	1,365,802	0.38	8
	WR100	6,007,510	87	8	1,175,173	0.33	4
	WR103	6,256,551	87	8	1,198,555	0.34	5
	WR111	3,899,236	88	7	894,765	0.25	5
	WR126	5,194,396	87	8	1,125,277	0.32	10
	WR133	6,545,660	87	8	1,227,038	0.35	4
	WR143	5,611,030	87	8	1,109,062	0.31	5
	WR171	3,852,279	88	7	966,368	0.27	6
	WR184	5,421,763	87	8	1,074,882	0.30	5
	WR195	5,985,028	87	8	1,168,776	0.33	6
	WR207	6,520,874	87	8	1,210,794	0.34	12
	WR219	6,951,983	87	8	1,312,291	0.37	6
	WR230	6,694,713	87	8	1,261,074	0.36	5
	WR233	3,623,110	88	7	876,065	0.25	4
	WR242	6,849,045	87	8	1,299,754	0.37	6
	WR256	5,991,356	87	8	1,119,055	0.32	6
	WR265	6,625,636	87	8	1,255,312	0.35	5
	WR280	6,621,409	87	8	1,249,681	0.35	5
	WR287	5,722,665	87	8	1,105,062	0.31	5

*Unique heterozygous SNP: SPNs that are uniquely present in one sample but absent in other samples.

### Genetic Differentiation of Wild Rice Populations

**FIGURE 3:**
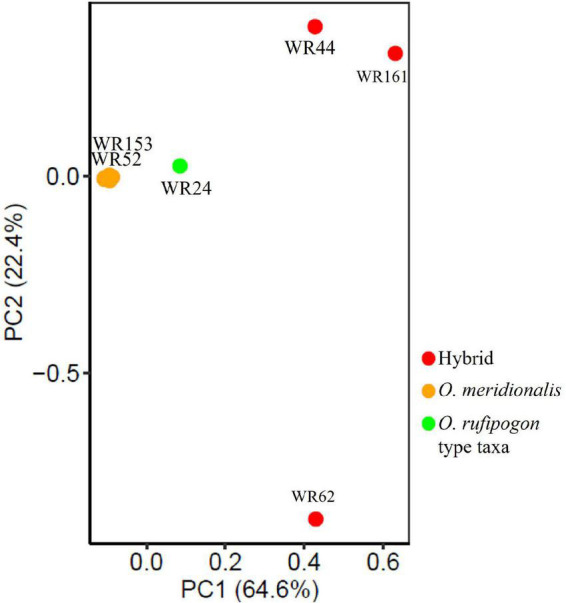
Principal component analysis (PCA) of 26 wild rice populations based on neutral 490,655 SNPs across the whole genome.

Genome-wide SNP data (12,247,414 SNPs) of 26 wild rice genotypes were first processed to remove linkage for PCA analysis. A total of 490,655 SNP sites were retained that fell below the linkage threshold. PCA was performed on linkage-pruned sites to determine the genetic groupings of wild rice populations. PCA analysis showed two clear clusters: (1) one group formed with hybrids (WR44, WR62, and WR161) and one putative parental taxon (WR24) and (2) all *O. meridionalis* populations along with two plausible later generations of hybrids (WR52 and WR153) formed another group (Figure 3). The first axis (PC1) explained 64.6% of the variation, which separated out the former group. There was a very low level of genetic differentiation observed in the latter group represented by the second axis (PC2) with 22.4% variation.

**FIGURE 4:**
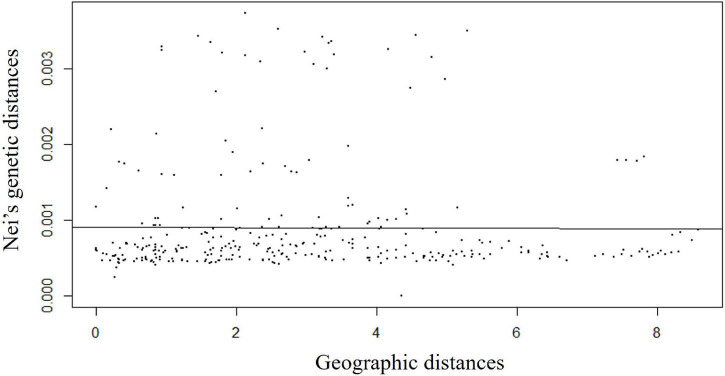
Isolation-by-distance (IBD) plots of Nei’s pairwise genetic distance values against the lineal geographic distance of Australian wild rice populations based on 2,987,364 SNPs and 26 sample sites.

Isolation by distance analysis based on a Monte-Carlo test with 999 permutations clearly showed that there was no significant relationship (*R*^2^ = −0.008, *p*-value = 0.42) between Nei’s pairwise genetic distance and geographic distances (Figure 4). A total of 300 pairwise Nei’s genetic distance data were plotted against pairwise geographic distance. The observed variation (*R*^2^) was 0.008 with a *p*-value of 0.42. This indicated genetic differentiation was not significantly increased with geographic distance, indicating genetic connectivity in wild rice populations across a wide range.

## Discussion

### Reticulate Origin of AA Genome Hybrids

This study confirms two types of the chloroplast genome in hybrid populations introgressed from one of their parental taxa. A total of five hybrids were studied of which only one hybrid (WR62) exhibited an *O. meridionalis* type chloroplast genome while the other four hybrids displayed *O. rufipogon* type chloroplast genomes. This indicates that hybridisation in both directions is possible. The previous study of these populations using the whole chloroplast and nuclear genome revealed chloroplast capture results in discordant cytonuclear phylogenies, inferring hybrid origins (Moner et al., 2018). Chloroplast capture can be the consequence of lineage sorting, convergent evolution, and recurrent hybridisation (Tsitrone et al., 2003). Unidirectional geneflow from recurrent hybridisation paves the way to capture either cytoplasm (Takahata and Slatkin, 1984) or nuclear genes (Petit et al., 2001) from one species to another species. However, introgression promotes chloroplast capture rather than nuclear genome capture in sympatric populations as has been detected in many plant species, resulting in discordant cytonuclear phylogenies (Rieseberg and Soltis, 1991). Unlike introgression, chloroplast capture can occur in species through one single hybridisation event and species with the freshly obtained chloroplast genome can be inherited by descendant lineages over time (Nge et al., 2021). In this study, hybridisation or introgressive hybridisation may have allowed chloroplast genome capture among sympatric wild rice populations. It is also known that nuclear genome incompatibilities may stimulate chloroplast capture in a random mating population (Tsitrone et al., 2003). Brozynska et al. (2017) reported that nuclear genomes of two parental taxa were distinguished where *O. rufipogon* type taxa exhibited more sequence similarity with *O. sativa* relative to the other Australian AA genome wild rice species. Whole nuclear genome analysis revealed that the Australian *O. rufipogon* had a nuclear genome that was part of a clade with Asian cultivated rice and its wild progenitors in Asia while *O. meridionalis* was sister to this clade in a nuclear genome phylogenetic tree (Moner et al., 2018). Despite the distinct nuclear genome, the chloroplast genome of the Australian *O. rufipogon* type taxa are more closely related to *O. meridionalis* differing by 53 variants and 36 variants from different accessions of *O. meridionalis*, respectively, while these forms of *O. meridionalis* from different regions (Queensland and Northern Territory) differed from each other by 34 variants (Brozynska et al., 2014). The *O. rufipogon* in Australia was much more divergent from *O rufipogon* in Asia (125 variants) and *O. sativa* spp. Nipponbare cv. *japonica* (125 variants). In the Australian *O. rufipogon*, ancient chloroplast capture has occurred from *O. meridionalis* (Brozynska et al., 2014), however, hybrids showed the two types of chloroplasts *(O. meridionalis* and Australian *O. rufipogon*) due to recent bidirectional chloroplast capture from these parents. The recent introgressive hybridisation demonstrated here may be an explanation of both the apparent chloroplast capture in the Australia *O. rufipogon* populations and the wild hybrids.

Analysis of nuclear genes demonstrated that the hybrids had alleles from both putative parental populations forming a heterozygote in early generations, indicating biparental nuclear geneflow from interspecific hybridisation. However, these loci become homozygous in what appear to be many later generations of hybrids that are fixed for the alleles of the recurrent parent, usually *O. meridionalis*. These observations clearly demonstrate an ongoing hybridisation and introgression persisting in the wild rice populations evidenced by the presence of viable hybrids between *O. rufipogon* type taxa and *O. meridionalis* being found at very low frequency. Earlier findings postulated that interspecific crossability between an *O. meridionalis* and a perennial Asian *O rufipogon* was modest but asymmetric (Banaticla-Hilario et al., 2013). As a result, they reported a very low percentage of fertile first filial generation (F1) (i.e., with mean F1 fertility = 17.2%) with perennial habit. However, they posited that a perennial habit enabled the F1 progeny to persist for a longer period than its annual counterpart. Like annual strains, crosses between accessions from the Jpn2 location (known to be *O. meridionalis*- like) and *O. rufipogon* produce low fertility pollen (<10%) as found with putative annual strains (<20–40%) (Sotowa et al., 2013). Intra-specific crossability between *O. meridionalis*, within the same geographical origin, produces fertile hybrids with pollen stainability (96.7%) and panicle fertility (66.9%), however, geographical distance seems to be the reason for the development of reproductive barriers (Juliano et al., 2005). They observed fertile hybrids, which were derived from crosses between Queensland accessions, however, crosses between accessions from different geographical regions produced sterile to partially fertile hybrids. Similarly, very low pollen stainability and seed fertility were reported in intraspecific crosses between *O. meridionalis* populations, from different geographical locations (Naredo et al., 1997).

The positive and negative significant *D*-statistic values from the four-taxon test indicate a nuclear geneflow from both putative parents into hybrids. Early generation hybrids exhibited higher heterozygosity and polymorphic variations than that of the later generations of hybrids. Repeated backcrossing between F1/F2 offspring and *O. meridionalis* may lead to comparatively low heterozygous SNP variation in the later-generation of hybrids. PCA analysis also reveal that early generation hybrids were clustered with *O. rufipogon* type taxa and later generation hybrids (except for WR161) form a group with *O. meridionalis*. Reproductive barriers underpin a termination of hybridisation and thereby, introduce introgression (Rieseberg and Wendel, 1993). An interspecific reproductive barrier gene (*SEED DEVELOPMENT 1*) on chromosome 6 is detected in *O. meridionalis* and *O. sativa* (Toyomoto et al., 2019). A recessive allele of a *SEED DEVELOPMENT 1* gene (*sdv1-m*) in wild rice strain from the Jpn2 site (=*O. meridionalis*) appears to evoke segregation distortion (1:2:0) in a BC_4_F_2_ progeny. Therefore, offspring carrying homozygous recessive alleles (*sdv1-m)* cause seed abortion at the seed development stage. Toyomoto et al. (2019) made an F1 offspring from a cross between *O. sativa* and *O. meridionalis*, then backcrossed with *O. sativa* to obtain backcrossing progeny. Despite homozygous recessive alleles, offspring bear seed if they are self-fertilised. *O. rufipogon* from Asia or Australia does not have this abortive gene. Backcrossing with *O. meridionalis* along with selfing may reduce seed abortion in later-generation hybrids.

Phenotypic characteristics of hybrids observed by Moner et al. (2018) seem consistent with the nuclear gene analysis. *O. rufipogon* type taxa are distinguished with open panicles and short awns from *O meridionalis*, which exhibits long awns with closed panicles. However, awn lengths sometimes overlapped (Brozynska et al., 2017; Moner et al., 2018). Early generation hybrids (WR44 and WR62) showed open to partially open panicles orientation like *O. rufipogon* type taxa. While all later generations hybrids exhibit panicles orientation like *O. meridionalis* type closed panicles. Interestingly, both generations of hybrids exhibited an intermediate anther length between two putative parental taxa and awn size close to *O. meridionalis*. Therefore, hybrids exhibit both intermediate and putative parental phenotypic characteristics, depending on which dominant loci of a trait are received from putative parents. Intermediate phenotypic characteristics have been observed in F1 hybrids derived from a cross between *O. rufipogon* and *O. meridionalis* (Naredo et al., 1997). Furthermore, F1 hybrids follow the lifecycle of their parental taxa, i.e., perennial F1 hybrids form when the two parental taxa are perennial in habit and annual and perennial parents produce semi-perennial to perennial offspring (Naredo et al., 1997). In contrast, Banaticla-Hilario et al. (2013) reported F1 offspring derived from a cross between Australasian perennial *O. rufipogon* and an annual *O. meridionalis* appears to be phenotypically closer to annual *O. meridionalis*. While F1 offspring with non-Australian *O. rufipogon* produce phenotypic characteristics more likely to be perennial *O. rufipogon* species. They also reported that interspecific F1 hybrids demonstrate either parental or intermediate phenotypic characteristics, i.e., some characteristics are analogous to their annual parental species and few features are intermediate between the two parents.

Reticulate evolution is a common phenomenon in vascular plants, wherein genetic changes occur through hybrid speciation, introgression, introgressive hybridisation, and horizontal gene transfer (Vriesendorp and Bakker, 2005). Hybrid origins were first proposed in the AA genome wild rice gene pool as an explanation of discordant cytonuclear phylogenies due to chloroplast capture (Moner et al., 2018). However, the disparity in cytonuclear phylogenies alone can never be an indicator of hybrid status (Vriesendorp and Bakker, 2005). Other events include incomplete lineage sorting, i.e., maintenance of ancestral polymorphisms through multiple speciation events (Andreasen and Baldwin, 2003; Goldman et al., 2004), homoplasy, and taxonomic sampling error (Wendel and Doyle, 1998) can cause such discordant patterns. The nuclear gene analysis reported here confirms hybrid events in the wild AA genome rice genepool and confirms that the discordant phylogenies are the result of hybridisation events. The nuclear gene analysis along with chloroplast genome data clearly reveal a reticulate evolution pattern in the wild AA genome rice gene pool.

*Oryza rufipogon* from other countries shows no evidence of hybridisation with *O. meridionalis* as this species is not found in other countries. No highly divergent AA genome species that *O. rufip*ogon could hybridise with is present outside Australia. This analysis is only possible in Australia where large populations of the two divergent species (*O. meridionalis* and the distinct Australian *O. rufipogon* like taxa) were searched to discover extremely rare apparent hybrids based upon their intermediate morphology. This study has confirmed the hybrid identity of these extremely rare plants. The Asian *O. rufipogon* samples that have been sequenced all show the presence of an *O. rufipogon* chloroplast that is distinct from that of *O. meridionalis* and the Australian *O. rufipogon* like taxa (Brozynska et al., 2014; Moner et al., 2018). The two AA genome species in Australia (*O. meridionalis* and the Australian *O. rufipogon* like taxa) remain as distinct populations despite the extremely rare hybridisation events between these two taxa confirmed in this study. The reproductive barrier between them resulting in low pollen fertility requires the backcrossing of the hybrids in subsequent generations.

### Australian Wild Rice Populations as a Reservoir for Novel Genetic Resources

This study reveals high levels of heterozygous SNP variation in AA genome wild rice populations, in relation to *O. sativa* ssp. *japonica* cv. Nipponbare. This finding is, in agreement with the result reported by Krishnan et al. (2014). They found that the Australian wild rice genepool contains a high level of genetic variations, in terms of, SNPs across the whole genome, i.e., 2,418,084 SNPs in *O. meridionalis* and 2,564,013 SNPs in *O. rufipogon*, compared to Asian *O. rufipogon* (917,738 SNPs) and cultivated *O. sativa* ssp. *indica* (978,630 SNPs), with respect to *O. sativa* ssp. *japonica* cv. Nipponbare. A molecular study using restriction fragment length polymorphism (RFLP) markers revealed that cultivated rice retained 40% of the wild alleles during rice domestication (Sun et al., 2001). This study reveals the level of heterozygosity is lower in *O. rufipogon* than in *O. meridionalis*. This is also consistent with the previous results reported by Brozynska et al. (2017). They postulated *O. meridionalis* (Taxon B) had a more heterozygous and repetitive genome than *O. rufipogon* (Taxon A), resulting in a higher number of scaffolds in the egnoem of this taxon. Mating systems play an important role in acquiring genetic variations. Perennials exhibit outcrossing mating system, which results in a higher degree of genetic variation than annuals while selfing is a feature of annuals (Oka, 1988). In Australia, *O. meridionalis* is common and widespread while the Australian *O. rufipogon* populations are much smaller. Comparison of the relative diversity of the two species in Australia will require more extensive sampling of the much rarer *O. rufipogon* populations. PCA and Isolation by-distance analysis also reveals a high degree of genetic connectivity among *O. meridionalis* populations across a wide geographic range in Northern Queensland, Australia. The role of animals (especially birds) in dispersing *Oryza* species over large distances is quite evident (Tang et al., 2010; Wambugu et al., 2015).

Natural variations in genes for agronomically important traits are limited in cultivated rice germplasm. In-plant breeding, desirable germplasm is sourced from wild species, landraces, and distant relatives, or for new traits through induced mutation or genetic manipulation (Ganeshan et al., 2010). The Australian wild rice populations include the earliest branching AA genome lineages and, therefore the most genetically and geographically distinct from the other AA genome wild relatives of cultivated rice. Asian domesticated rice was introduced to Australia around 200 years ago and mainly grown far from wild rice populations (Henry et al., 2010) avoiding genetic introgression of domestication genes into the wild populations. Recent studies already reveal some interesting features of the native Australian populations, in terms of disease resistance, grain appearance, and nutritional properties (Kasem et al., 2012, 2014; Tikapunya et al., 2017) suggesting that they could be exploited to extract numerous genes for agronomically important traits for rice improvement.

## Data Availability Statement

The datasets presented in this study can be found in online repositories. The names of the repository/repositories and accession number(s) can be found below: https://www.ncbi.nlm.nih.gov/genbank/, BioProject number PRJNA758754.

## Author Contributions

RH conceived the project. AF and RH collected field samples. SH, AF, and RH analysed the data. SH wrote the first draft of the manuscript. All authors contributed to the article and approved the submitted version.

## Conflict of Interest

The authors declare that the research was conducted in the absence of any commercial or financial relationships that could be construed as a potential conflict of interest.

## Publisher’s Note

All claims expressed in this article are solely those of the authors and do not necessarily represent those of their affiliated organizations, or those of the publisher, the editors and the reviewers. Any product that may be evaluated in this article, or claim that may be made by its manufacturer, is not guaranteed or endorsed by the publisher.

## Footnotes

1
http://www.clcbio.com

2
https://www.geneious.com

## Supplementary Material

Supplementary Figure 1 The phylogenetic tree of the chloroplast genome of four samples and two reference chloroplast genomes (*O. meridionalis* and *O. rufipogon* type taxa), with *O. officinalis* as the outgroup. Among four samples, WR62 and WR161 (red in colour) were taken from this study while the other two samples WR65 and WR162 (green in colour) were taken from Moner et al. (2018). The tree was generated using the maximum likelihood (ML) method in PAUP* version 4 software with 1,000 bootstrap replicates. The ML bootstrap value (/40) is marked on each node.
