# Supplementary figures and images for: Reticulate Evolution in AA-Genome Wild Rice in Australia
Source: Front Plant Sci. 2022 Mar 11;13:767635. doi: 10.3389/fpls.2022.767635 (PMC8963485; doi:10.3389/fpls.2022.767635)

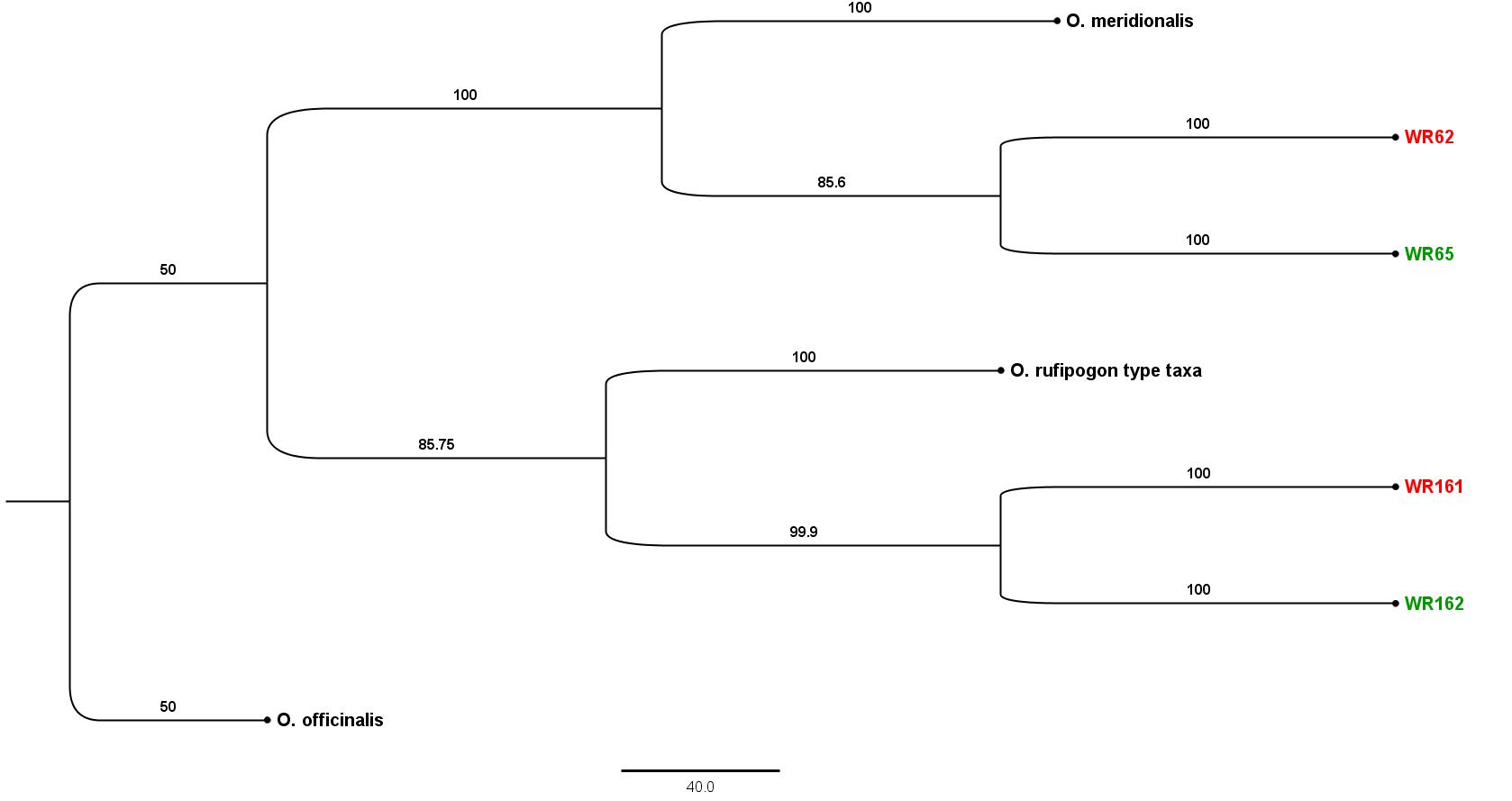

Supplement: Supplementary Figure 1 — The phylogenetic tree of the chloroplast genome of four samples and two reference chloroplast genomes (O. meridionalis and O. rufipogon type taxa), with O. officinalis as the outgroup. Among four samples, WR62 and WR161 (red in colour) were taken from this study while the other two samples WR65 and WR162 (green in colour) were taken from Moner et al. (2018). The tree was generated using the maximum likelihood (ML) method in PAUP* version 4 software with 1,000 bootstrap replicates. The ML bootstrap value (/40) is marked on each node. [file Image_1.JPEG]
