# Supplementary material for: Reticulate Evolution in AA-Genome Wild Rice in Australia
Source: Front Plant Sci. 2022 Mar 11;13:767635. doi: 10.3389/fpls.2022.767635 (PMC8963485; doi:10.3389/fpls.2022.767635)
Supplement: Supplementary file 2 [file Table_1.DOCX]

| Wild rice accession | Total number of trimmed  reads | Total number of trimmed  nucleotides | Average sequence read depth (times reference length) | Average mapped sequence read depth (times reference length) | Mapped reads (%) | Mapped bases (%) | Total Consensus length (bp) | Total consensus as % of reference |
| --- | --- | --- | --- | --- | --- | --- | --- | --- |
| WR24 | 98,924,768 | 14,260,593,039 | 37.32 | 8.88 | 23.98 | 23.79 | 322,074,565 | 81.96 |
| WR44 | 79,543,302 | 11,329,429,070 | 29.65 | 21.31 | 71.98 | 71.87 | 317,197,054 | 83.00 |
| WR52 | 91,892,853 | 13,174,543,743 | 34.47 | 15.59 | 45.45 | 45.21 | 266,474,097 | 69.73 |
| WR81 | 96,647,340 | 13,826,809,815 | 36.18 | 25.00 | 69.15 | 69.09 | 274,917,517 | 71.94 |
| WR103 | 95,866,473 | 13,859,437,006 | 46.92 | 46.70 | 46.87 | 46.7 | 269,178,161 | 70.44 |
| WR111 | 83,267,631 | 11,884,028,952 | 31.10 | 9.61 | 31.14 | 30.91 | 260,345,610 | 68.13 |
| WR133 | 72,753,336 | 10,352,724,630 | 27.09 | 18.49 | 68.33 | 68.26 | 270,764,352 | 70.85 |
| WR 207 | 79,244,286 | 11,204,692,001 | 29.32 | 19.45 | 66.51 | 66.32 | 268,579,423 | 70.28 |
| WR 37 | 77,206,124 | 11,323,300,556 | 29.63 | 8.96 | 30.52 | 30.25 | 257,716,289 | 67.44 |
| WR 62 | 79,172,066 | 11,450,793,351 | 29.96 | 14.59 | 48.91 | 48.69 | 313,650,369 | 82.07 |
| WR 100 | 79,922,587 | 11,616,872,760 | 30.40 | 15.44 | 51.00 | 50.78 | 266,834,329 | 69.82 |
| WR126 | 60,113,679 | 8,704,460,829 | 22.78 | 12.54 | 55.23 | 55.04 | 263,722,779 | 69.01 |
| WR 143 | 56,144,819 | 8,183,989,092 | 21.42 | 13.59 | 63.53 | 63.45 | 263,407,095 | 68.93 |
| WR153 | 54,631,067 | 7,925,007,230 | 20.74 | 13.97 | 67.39 | 67.36 | 262,563,254 | 68.71 |
| WR161 | 69,269,233 | 10,105,816,105 | 26.44 | 15.74 | 59.65 | 59.53 | 266,028,616 | 69.61 |
| WR 171 | 75,698,012 | 11,057,490,330 | 28.93 | 11.06 | 38.45 | 38.21 | 258,379,810 | 67.61 |
| WR184 | 65,726,306 | 9,580,839,457 | 25.07 | 13.44 | 53.82 | 53.61 | 262,299,170 | 68.64 |
| WR195 | 65,675,525 | 9,450,951,461 | 24.73 | 15.50 | 62.78 | 62.66 | 267,892,702 | 70.10 |
| WR219 | 84,313,296 | 12,275,734,508 | 32.12 | 21.77 | 67.83 | 67.76 | 269,530,363 | 70.53 |
| WR230 | 75,856,818 | 11,038,295,474 | 28.88 | 20.48 | 70.93 | 70.89 | 268,379,182 | 70.23 |
| WR233 | 75,798,242 | 10,936,594,201 | 28.62 | 9.25 | 32.47 | 32.31 | 257,764,957 | 67.45 |
| WR 242 | 77,978,446 | 11,394,843,826 | 29.82 | 21.29 | 71.43 | 71.41 | 270,190,325 | 70.70 |
| WR256 | 64,971,673 | 9,455,903,833 | 24.74 | 15.62 | 63.11 | 63.12 | 262,514,763 | 68.69 |
| WR265 | 75,186,237 | 10,811,748,409 | 28.29 | 19.57 | 69.22 | 69.16 | 271,348,121 | 71.01 |
| WR280 | 71,628,994 | 10,436,216,009 | 27.31 | 18.93 | 69.34 | 69.32 | 269,112,327 | 70.42 |
| WR287 | 75,036,673 | 10,942,077,255 | 28.63 | 14.22 | 49.87 | 49.67 | 264,573,225 | 69.23 |

Table S1. Summary statistics of whole genome sequencing of 26 wild rice samples and mapping to O. sativa spp. japonica cv. Nipponbare as reference.
