# Supplementary material for: Reticulate Evolution in AA-Genome Wild Rice in Australia
Source: Front Plant Sci. 2022 Mar 11;13:767635. doi: 10.3389/fpls.2022.767635 (PMC8963485; doi:10.3389/fpls.2022.767635)
Supplement: Supplementary file 3 [file Table_2.DOCX]

Table S2: Mapping of sequences from 8 wild rice taxa to *O. rufipogon* type draft genome, *O. meridionalis* draft genome and Os-Nipponbare-Reference-IRGSP-1.0 reference genome.

|  | - 1. trimmed data | | *O. rufipogon* type taxa draft genome | | | *O. meridionalis* draft genome | | | Os-Nipponbare-Reference-IRGSP-1.0 | | |
| --- | --- | --- | --- | --- | --- | --- | --- | --- | --- | --- | --- |
| Sample ID | Total reads | Total nucleotides | Average sequence read depth (times of reference length) | Average mapped sequence read depth (times of reference length) | Total consensus as % of reference | Average sequence read depth (times of reference length) | Average mapped sequence read depth (times of reference length) | Total consensus as % of reference | Average sequence read depth (times of reference length) | Average mapped sequence read depth (times of reference length) | Total consensus as % of reference |
| WR24 | 98924768 | 14260593039 | 37.06 | 9.47 | 94.92 | 40.18 | 8.11 | 71.25 | 37.32 | 8.88 | 81.96 |
| WR44 | 79543302 | 11329429070 | 29.45 | 21.82 | 87.81 | 31.92 | 23.67 | 89.53 | 29.65 | 21.31 | 83.00 |
| WR52 | 91892853 | 13174543743 | 34.24 | 15.38 | 68.83 | 37.12 | 20.88 | 88.55 | 34.47 | 15.59 | 69.73 |
| WR81 | 96647340 | 13826809815 | 35.94 | 24.67 | 71.31 | 38.96 | 33.76 | 87.76 | 36.18 | 25.00 | 71.94 |
| WR103 | 95866473 | 13859437006 | 36.02 | 16.72 | 69.73 | 39.05 | 22.52 | 88.52 | 46.92 | 46.70 | 70.44 |
| WR111 | 83267631 | 11884028952 | 30.89 | 9.49 | 67.20 | 33.48 | 12.80 | 87.77 | 31.10 | 9.61 | 68.13 |
| WR133 | 72753336 | 10352724630 | 26.91 | 18.27 | 70.30 | 29.17 | 25.27 | 90.30 | 27.09 | 18.49 | 70.44 |
| WR 207 | 79244286 | 11204692001 | 29.12 | 19.14 | 69.22 | 31.57 | 25.47 | 87.48 | 29.32 | 19.45 | 68.13 |
